# Nutritional status of children with neurodevelopmental disorders: a cross-sectional study at a tertiary-level hospital in northern Bangladesh

**DOI:** 10.1186/s40795-024-00863-9

**Published:** 2024-04-19

**Authors:** Rabeya Khatun, Md. Kaoser Bin Siddique, Mst. Reshma Khatun, Maskura Benzir, Md. Rafiqul Islam, Sohel Ahmed, Olav Muurlink

**Affiliations:** 1 Department of Pediatrics, TMSS Medical College & Rafatullah Community Hospital (TMC&RCH), Bogura, Bangladesh; 2 Research, Planning & Development (RP&D), TMSS Grand Health Sector (TGHS), TMSS, Rangpur Road, Thengamara,, Bogura, Bangladesh; 3 Department of Pharmacy, Manarat International University, Dhaka, Bangladesh; 4 Department of Anatomy, TMSS Medical College (TMC), Rangpur Road, Thengamara, Bogura, Bangladesh; 5 Ahmed Physiotherapy & Research Center, Kalabagan, Dhaka, Bangladesh; 6 Sustainable Innovation, School of Business and Law, Central Queensland University, Brisbane, Australia

**Keywords:** Children, Nutritional status, Malnutrition, Disability, Neurodevelopmental disorders, Bangladesh

## Abstract

**Background:**

Malnutrition in children with neurodevelopmental disorders (NDDs) is a significant global public health issue. Nutritional assessment combined with management or advice are essential to produce optimal outcomes.

**Objectives:**

The objective of this study was to assess nutritional status and the sociodemographic profile of children with neurodevelopmental disorders in Bangladesh.

**Methods:**

A cross-sectional study was conducted from December to April 2020 among the population of children with NDDs who presented to the pediatric department of the TMSS Medical College and Rafatullah Community Hospital in Bogura during this period. Socio-demographic data along with anthropometric measurements of the children were taken. Assessment of nutritional status were made using metrics such as *z*-scores for weight-for-age (WAZ), height-for-age (HAZ), and body mass index-for-age (BAZ). Descriptive statistics (number and percentage) and analytical statistics (*chi*-square and logistic regression) were included.

**Results:**

58.6% of children displayed malnutrition, with 47.8% showing undernutrition (WHZ / BAZ − 1 SD-≤-3 SD), and 10.8% overnutrition (BAZ > 2SD). Significant negative associations were found between malnutrition and parental education level, urban residency, and monthly family income. Children diagnosed with cerebral palsy exhibited twice the likelihood to be malnourished (AOR 2.39, 95% CI 0.83–6.87). Furthermore, residing in rural regions was associated with an increased risk of experiencing malnutrition, as indicated by an adjusted odds ratio of 1.60 (95% CI 0.12–3.09).

**Conclusions:**

While the results are cross-sectional, over half of children with NDDs were found to be malnourished, suggesting that children with NDD in Bangladesh are vulnerable to developing any form of malnutrition. Therefore, regular assessments and timely nutritional support may improve their situation.

**Supplementary Information:**

The online version contains supplementary material available at 10.1186/s40795-024-00863-9.

## Introduction

Malnutrition in children with neurodevelopmental disorders (NDDs) is a global public health issue. Children with NDDs are typically very prone to malnutrition, either under- or over-nutrition [1] both of which are associated with negative health consequences. Adequate nutrition is independently associated with basic health outcomes, including survival and quality of life [1, 2]. According to the Diagnostic and Statistical Manual of Mental Disorders (Fifth Edition) neurodevelopmental disorders are described as “a group of conditions with onset in the developmental period. The disorders typically manifest early in development, often before the child enters [formal schooling], and are characterized by developmental deficits that produce impairments of personal, social, academic, or occupational functioning” [3]. Disability is neither exclusively biological nor social in origin or manifestation; rather, it results from the combination of personal and environmental variables [4]. Globally 15.6% of people have a disability, and an alarming 80% of those living with a disability present in low- and middle-income countries (LMICs) [5]. The prevalence of disability in Bangladesh is 15.6% [6] but the prevalence of malnutrition among children in this category remains unclear.

Malnutrition remains one of the leading causes of childhood mortality in LMICs, and complications emerge when malnutrition is coupled with a disability [7]. Malnutrition accounts for over half of infant and child deaths worldwide each year, and 3.1 million children with disabilities die from malnutrition [7]. Children with disabilities are three times more likely to be underweight than typically developed children [8]. Even in developing countries, the actual prevalence of malnutrition and its consequences vary significantly. In Kenya, disabled children are 2.2 and 1.7 times more likely to be underweight and stunted, respectively, than their age- and gender-matched healthy peers [9]. By contrast, in Bangladesh, a previous study suggested these children are 6.6 times higher odds of being severely underweight and 11.8 times higher odds of being severely stunted [10]. These results suggest that sociocultural differences may be amplifying malnutrition.

An influential framework described by Groce et al. shows that malnutrition may occur in children with NDDs when there is an increased need for nutrients, greater nutrient loss, and decreased nutrient intake [11]. Malnutrition is also associated with a child’s growth and development; it reduces peripheral blood circulation, which causes delayed wound healing and increases spasticity and irritability [12]. As for the third aspect of the Groce model, reduced intake, malnutrition resulting from inadequate parental care affects 70% of neurologically challenged children residing in residential settings, placing them at risk for stunted growth, inadequate nutrition, and delayed physical and mental development [12].

There have been limited studies examining the interaction between malnutrition and disability in Bangladesh. A study focusing on children with permanent disabilities paired with age-matched controls found that severe acute malnutrition was four times as likely among children with disability, and the prevalence of underweight and stunting in children with CP was 71.2% and 75.0%, respectively, and in children with epilepsy it was 75.9% and 79.4%, respectively [10]. A study examining malnutrition in Bangladeshi children with cerebral palsy found that more than two-thirds of the sample of 726 were underweight, with moderate to severe undernutrition significantly associated with sociodemographic factors such as age, monthly family income, and physiological factors including gross motor function and neurological type [13]. The current study adds to this small body of work, offering the strength of a hospital-based study with physician-conducted diagnoses, and using exclusion criteria that allows the study to examine the role played by associated factors other than the disability in contributing to malnutrition. In particular, the study illuminates the role of socioeconomic factors in driving malnutrition in developing world children with a disability, so that a proper nutritional assessment, support management can be designed for children and their caregivers.

## Methods

### Study design, setting and sample selection

This descriptive cross-sectional study was conducted from December 2020 to April 2022 at TMSS Medical College Hospital and Rafatullah Community Hospital, Bogura, at the Department of Pediatrics and Child Development Center. The facilities include outdoor/outpatient, clinical wards, and rehabilitation centre/services. Patients are normally assessed in the outdoor/outpatient department, and referred either to the hospital or the rehabilitation centre. In this case, patients who met the sample selection criteria (see below) and were referred to the rehabilitation centre (*not* the hospital) were taken as the study population. Parents and legal guardians of children with NDDs were interviewed through a pretested structured questionnaire, and anthropometric measurements were taken of each child to assess nutritional status.

### Sample selection criteria

- **Inclusion Criteria**: Only children with NDD diagnoses independently confirmed by a physician, within the age range of 6 to 144 months, accompanied by parents who were able and willing to participate and provide written consent were included in the study. Only children accepted into the rehabilitation centre, not the associated hospital, were included.
- **Exclusion criteria**: Children with known chronic ailments affecting nutrition like congenital heart disease, chronic renal disease, human immunodeficiency virus (HIV) infection, and other congenital anomalies that would independently affect their food intake, e.g., cleft lip and cleft palate, were excluded from the study.

According to the Neuro Developmental Disorder (NDD) Protection Trust Act 2013, NDD comprises four conditions: Autism Spectrum Disorder (ASD), Cerebral Palsy (CP), Down Syndrome (DS) and 4. Intellectual Disability (ID). However, this study used six additional conditions under NDD: CP with epilepsy; epilepsy; DS with epilepsy; Attention-deficit/hyperactivity disorder (ADHD); speech delay and developmental delay. Note that none of the sample exhibited DS with epilepsy.

### Sample size estimation

The sample size has been calculated by applying the single population proportion formula, under the assumptions of a 95% level of significance, a 5% margin of error, and a response distribution of 50% were used to calculate the sample size. The following calculation, where n = sample size, p = prevalence, and d = margin of error, was used to determine the necessary sample size:

N = z^2^ p (1-p) / d^2^, n = (1.96)^2^ × (0.5) (0.5) / (0.03)^2^, = 384.

However, the challenges posed by the COVID-19 pandemic, including lockdown measures and time limitations, made it arduous to gather data and identify children with neurodevelopmental disorders (NDD) as responders. Consequently, the research team had difficulties in locating 384 participants within the designated timeframe of our research. Consequently, there was a need to increase the margin of error from 5 to 8%, necessitating a final sample size of 150. In light of an anticipated 5% rate of incomplete responses, the final minimum needed sample size for this research has been determined to be 157.

### Survey tool development

A questionnaire was developed based on the objectives of this study. The Gross Motor Function Classification System (GMFCS) developed by CanChild in Canada was used as a basis for the questionnaire. The questionnaire was translated into Bangla language for ease of comprehension in a population that includes the full spectrum of education levels.

[Survey Questionnaire as supplementary file]

- ***Parents’ socio-demographic variables***: Demographic characteristics such as level of education, occupation, area of residency (rural/urban), family size, and monthly family income were included.
- ***Demographic variable of children***: Demographic variables including age, sex, and clinical diagnosis, weight, height and BMI were included.

### Outcome variables

The criteria developed by WHO (2009) for the classification of acute malnutrition in children was employed [14]. The WHO recommends using the formula z-score = (X-m)/SD, where X is the observed value (height, weight, or BMI), m is the mean value, and SD is the value of the standard deviation of the distribution that corresponds to the reference population.

Weight for height, BMI for age, and height for age comparisons were used to establsh wasting, which means acute malnutrition, and stunting, that is, chronic malnutrition, respectively [15]. According to the Z score classification, weight-for-height z-score WHZ/BAZ ≤-3 SD indicates acute severe malnutrition; -2.99 to -1 SD Mild to moderate malnutrition and overnutrition were indicated by ˃2 SD.

### Data collection and management

Clearance was obtained from hospital authorities, and informed written consent was obtained from each parent or guardian at the time of the interview. Detailed information was collected using a structured, pretested standard questionnaire by the face-to-face interview method. Children with a diagnosis of NDD made by a medical doctor and confirmed by patient records attending the pediatrics department and Child Development Center (Shishu Bikash Kendro) were recruited for the study. Children previously identified as having a disability underwent a detailed clinical assessment to confirm the diagnosis. The measures included in the instrument were presented orally to parents or guardians in the Bangla language. Anthropometry measurements were conducted by clinicians or nurses, with the weight and height of each study participant recorded according to the standard guidelines of the World Health Organisation [16]. Each child’s weight was measured to the nearest 0.1 kg using a baby scale for children under one year and a digital bathroom scale for the rest of the children. Children unable to stand were measured in their parent’s or guardian’s arms. The difference between the combined weight of the bearer of the child and child and that of the bearer alone was recorded as the child’s weight. Height was measured using a stadiometer for children older than 2 years who were able to stand flat-footed and straight. The recombinant length of the participants less than 2 years of age and those who were 2 years of age or older and were unable to stand upright was measured using an infantometer with a fixed head and a movable footboard. For children with physical deformities who were unable to stand independently, skeletal contracture segmental measurements are used to estimate stature. The segmental measurements are upper-arm length and tibial length. These approaches are considered valid for measuring the stature of children with cerebral palsy. In this study, tibial length was preferred. We utilized the equation for estimating stature in CP by Stevenson et al. [17].

All z scores were calculated using WHO Anthro and WHO Anthro Plus software. Then nutritional status was defined by WHO standards based on the z scores. Height for age and weight for height was used for stunting and wasting respectively [16]. 

### Survey validation and administration

The draft of the survey questionnaire was not subjected to content validation, as we used a WHO-validated tool [18]. The final draft of the questionnaire was piloted initially on five young patients who would otherwise have been qualified for the full study, to check the response time. Piloting suggested it took around 20 min to complete the questionnaire. Children with neurodevelopmental disorders (NDDs) who were regularly attending the out-patient department (OPD), and in-patient department (IPD) of the paediatrics department, and child development centre of TMSS Medical College and Rafatullah Community Hospital, Bogura. The patient’s medical records and clinical examination were used to verify their diagnosis.

### Statistical analysis

Data was first logically sequenced using Microsoft Excel for Windows. Data analysis was carried out using the software SPSS 20.0 (IBM Corporation, New York, USA) for Windows. The continuous variables are presented as the mean and a 95% confidential interval. Categorical variables are presented as numbers and percentages. The Pearson chi-square test or Fisher’s exact test was used to find out the association between variables. Binary logistic regression was used to assess the predicted risk factors for nutritional status, using odds ratio (OR) and a 95% confidence interval (CI). The level of significance was set at < 0.05.

## Results

### Demographics of the child with NDDs and their parents

A total of 157 children (57.3% male) with neurodevelopmental disorders took part in this study. The mean age of the participants was 63.68 months, ranging in age from six to 144 months. Cerebral palsy was most common: 27.4% of children were diagnosed with cerebral palsy, with a further 24.8% of children diagnosed as suffering from cerebral palsy with epilepsy. In this study, about 28% of parents had no formal education, 33.4% of parents’ occupation was listed as a business, most of the participants (68.8%) were from rural areas, and 42.7% of participants’ gross monthly family income was < 10,000 Bangladeshi taka^1^. Nearly half of the participants about 47.1% of participants had 4–5 household members. The details are presented in Table 1.

**Table 1:** Bio-demographic status of children with NDD and their parents (*n* = 157)

Variable					Number			Percentage	
Age in months									
< 24					33			21	
25–48					42			26.8	
49–72					27			17.2	
73–96					15			9.6	
97–120					24			8.9	
> 120					26			16.6	
Total					157			100	
Mean age					63.68				
Range					6-144				
SD					42.95				
Sex									
Male					90			57.3	
Female					67			42.7	
Total					157			100	
Clinical diagnosis									
Cerebral palsy					43			27.4	
Cerebral palsy with epilepsy					39			24.8	
Epilepsy					10			6.4	
Down syndrome					11			07	
Autism spectrum disorder					21			13.4	
ADHD					05			3.2	
Speech delay					15			9.6	
Developmental delay					10			6.4	
Intellectual disability					03			1.9	
Total					157			100	
Residence									
Urban					37			23.6	
Semi-urban					12			7.6	
Rural					108			68.8	
Total					157			100	
Monthly Family income (BDT/USD)									
< 10,000 (< 101 USD)					67			42.7	
11,000–25,000 (102–240 USD)					46			29.3	
26,000–35,000 (241-342USD)					16			10.2	
≥ 35,000 (≥ 342USD)					28			17.8	
Total					157			100	
Family type									
Nuclear					109			69.4	
Extended					48			30.6	
Household size									
Small Family 1–3 Members					28			17.8	
Medium Family4-5 Members					78			47.1	
Large Family ≥ 6 Members					55			35	
Total					157			100	
	**Father**						**Mother**		
	**Number**		**Percentage**				**Number**		**Percentage**
Education									
No formal Education		31		19.7		13			8.3
Up to Primary		26		16.6		24			15.3
Below SSC		30		19.1		48			30.6
SSC		22		14		32			20.4
HSC		11		7		14			8.9
Bachelors		16		10.2		12			7.6
Masters and above		21		13.4		14			8.9
Total		157		100		157			100
Occupation									
Govt. Service		7		4.5		7			4.5
Other Service		20		12.7		5			3.2
Business		51		32.5		3			1.9
Day labor		14		8.9		01			0.6
Farmer		35		22.3		-			-
Housewife		-		-		135			86
Unemployed		01		0.6		-			-
Others		29		18.5		06			3.8
Total		157		100		157			100

### Nutritional status of the children

Of the total sample, 47.8% exhibited wasting with 8.9% of the total sample exhibiting severe wasting (BAZ ≤-3 SD) and the balance had mild to moderate wasting (BAZ − 2.99 to -1 SD). On the other hand, 10.8% of children were overweight (BAZ ˃2 SD). 57.3% of children in the sample were stunted and the remaining 42.7% of had age-appropriate height (HAZ − 0.99 to 1.99 SD). The nutritional status of sample is presented in Table 2 with the disease-specific nutritional status of the child presented in Table 3.

**Table 2:** Nutritional status of the study population (*n* = 157)

Nutritional indices reported as Z score		
Categories	Weight for height (WHZ) / BMI for age Z score (BAZ)	Children’s height/length for age Z score (HAZ)
Z Score	f (%)	f (%)
≤-3 SD	14 (8.9)	29 (18.5)
-2.99 to -1 SD	61(38.8)	61 (38.8)
-0.99 to 1.99 SD	65(41.4)	67(42.7)
˃2 SD	17(10.8)	-
Total	157(100%)	157(100%)

**Table 3:** Nutritional status and children’s clinical diagnosis (*n* = 157)

variables	Nutritional status		x2	*P* value	Nutritional status	x2	*P* value
	Wasting	Over weight			Stunting		
Cerebral palsy (CP)	26 (34.7%)	02 (11.8%)	38.052	0.001	29 (32.2%)	25.533	0.001
Cerebral palsy with Epilepsy	24 (32%)	02 (11.8%)			28 (31.1%)		
Epilepsy	06 (8%)	0			06 (6.7%)		
Down syndrome	02 (2.7%)	02 (11.8%)			09 (10%)		
Autism Spectrum Disorder (ASD)	04 (5.3%)	04 (23.5%)			06 (6.7%)		
ADHD	01 (1.3%)	03 (17.6%)			0		
Speech delay	08 (10.7%)	01 (5.9%)			05 (5.6%)		
Developmental Delay	02 (2.7%)	02 (11.8%)			05 (5.6%)		
Intellectual Disability	02 (2.7%)	01 (5.9%)			02 (2.2%)		
Total	75 (100%)	17 (100%)			90 (100%)		

### Association between the nutritional status of child and socio-demographic of their parents

There was a significant association between the nutritional status of children and their parents’ education level, in particular that of the mother (father, *p* = 0.019 and mother, *p* = 0.003) as well as with the father’s occupation (*p* = 0.022). Findings also reveal a highly significant association between the nutritional status of children and the family’s monthly income (p = < 0.001). Children from families in rural areas (*p* = 0.042) and children from families with income below 10,000 taka (*p* < 0.001) were significantly more likely to be malnourished. Details are presented in Table 4.

**Table 4:** Association between nutritional status and socio-demographic status of children with NDDs (*n* = 157)

	Variables	Father		X2	*P* value	Mother		X2	*P* value
		Nutritional status				Nutritional status			
		Wasting	Over weight			Wasting	Over weight		
	No formal education	18 (24%)	2 (11.8%)	24.24	0.019	06 (8%)	01 (5.9%)	29.81	0.003
	Up to primary	15 (20%)	02 (11.8%)			14 (18.7%)	0		
Education level	Below SSC	15 (20%)	0			28 (37.3%)	04 (23.5%)		
	SSC	15 (20%)	02 (11.8%)			21 (28%)	03 (17.6%)		
	HSC	03 (4%)	02 (11.8%)			03 (4%)	03 (17.6%)		
	Bachelors	05 (6.7%)	04 (23.5%)			01 (1.3%)	02 (11.8%)		
	Masters and above	04 (5.3%)	05 (29.4%)			02 (2.7%)	04 (23.5%)		
	Total	75 (100%)	17 (100%)			75 (100%)	17 (100%)		
Occupation	Govt. Service	01 (1.3%)	0	23.77	0.022	01 (1.3%)	0	15.43	0.117
	Other Service	04 (5.3%)	06 (35.3%)			02 (2.7%)	02(11.8%)		
	Business	27 (36%)	07 (41.2%)			01 (1.3%)	0		
	Day labor	09 (12%)	01 (5.9%)			01 (1.3%)	0		
	Farmer	16 (21.3%)	02 (11.8%)			-	-		
	Housewife	-	-			65 (86.7%)	15(88.2%)		
	Unemployed	01 (1.3%)	0			-	-		
	Others	17 (22.7%)	01(5.1%)			5 (6.7%)	0		
	Total	75 (100%)	17 (100%)			75 (100%)	17(100%)		
		Nutritional status							
		Wasting		Over weight					
Residence	Urban	11 (14.7%)		07 (41.2%)				8.71	0.042
	Semi-urban	06 (8%)		0					
	Rural	58 (77.3%)		10 (58.8%)					
	Total	75 (100%)		17 (100%)					
Family income (BDT)	≤ 10,000	41 (54.7%)		03 (17.6%)				27.34	0.000
	11,000–25,000	23 (30.7%)		02 (11.8%)					
	26,000–35,000	02 (2.7%)		06 (35.3%)					
	≥ 35,000	09 (12%)		06 (35.3%)					
	Total	75 (100%)		17 (100%)					
Family type	Nuclear	57 (76%)		09 (52.9%)				4.03	0.133
	Extended	18 (24%)		08 (47.1%)					
Household size	Small Family 1–3 Members	12 (16%)		03 (17.6%)				3.77	0.438
	Medium Family 4–5 Members	41 (54.7%)		06 (35.3%)					
	Large Family ≥ 6 Members	22 (29.3%)		08 (47.1%)					
	Total	75 (100%)		17 (100%)					

### Result of regression analysis

Prior to conducting binary logistic regression, we assessed the presence of multicollinearity among the independent variables through the utilization of the Variance Inflation Factor (VIF). The VIF values of all variables were less than 3 suggesting there was no issue with multicollinearity. In the unadjusted model, Age; Father’s Education; Father’s Occupation were significantly associated with malnutrition among the NDD children whereas the adjusted model revealed only significant association between child’s age and malnutrition. Children diagnosed with Cerebral Palsy exhibit twice as great a likelihood to be susceptible to malnourishment (AOR 2.39, 95% CI 0.83–6.87, P ˂ 0.001). Furthermore, residing in rural regions is associated with an increased risk of experiencing malnutrition, as indicated by an adjusted odds ratio of 1.60 (95% CI 0.12–3.09). Children aged in categories greater than 25 months (25–48, 49–72, 73–96, 97–120, and > 120 months) all had a greater chance of being malnourished with odds ratios of (AOR 4.70, 95% CI 1.55–14.27, *p* = 0.006), (AOR 4.74, 95% CI 1.37–16.32, *p* = 0.014), (AOR 5.61, 95% CI 1.29–24.29, *p* = 0.014), (AOR 4.65, 95% CI 0.99–21.66, *p* = 0.050), and (AOR 4.20, 95% CI 1.26–13.97, *p* = 0.019) respectively than children aged at or below 24 months. Details are presented in Table 5.

**Table 5:** Factors associated with malnutrition among the 6 to 144-month-olds NDD children (Binary logistic regression analysis)

Characteristics	Unadjusted OR (95% CI)	*p*-values	Adjusted OR* (95% CI)	*p*-values
Age (month)				
< 24 (ref)	1			
25–48	3.15 (1.22–8.14)	0.018*	4.70 (1.55–14.27)	0.006*
49–72	2.97 (1.03–8.54)	0.043*	4.74 (1.37–16.32)	0.014*
73–96	4.81 (1.25–18.50)	0.022*	5.61 (1.29–24.29)	0.021*
97–120	2.33 (0.65–8.34)	0.192	4.65 (0.99–21.66)	0.050*
> 121	3.30 (1.13–9.69)	0.029*	4.20 (1.26–13.97)	0.019*
Father education				
Masters and above (ref)	1			
Bachelors	1.71 (0.46–6.37)	0.421	2.00 (0.42–9.50)	0.383
HSC	1.11 (0.25–4.82)	0.888	0.61 (0.09–4.18)	0.620
SSC	4.53 (1.21–16.96)	0.025*	3.32 (0.51–21.64)	0.210
Below SSC	1.33 (0.43–4.09)	0.615	0.73 (0.13–3.97)	0.713
Up to Primary	2.52 (0.77–8.22)	0.126	1.26 (0.20–7.92)	0.802
No formal Education	2.42 (0.78–7.54)	0.126	1.53 (0.26–8.95)	0.637
Father occupation				
Service (ref)	1			
Business	2.91 (1.11–7.62)	0.030*	2.34 (0.67–8.12)	0.181
Day labor	2.01 (0.77–5.20)	0.151	0.91 (0.19–4.37)	0.910
Others	2.38 (0.81–6.96)	0.113	1.18 (0.25–5.57)	0.834
Household Income				
> 35,000 Taka (ref)	1			
26,000–35,000 Taka	0.87 (0.25–2.96)	0.819	0.72 (0.16–3.27)	0.716
11,000–25,000 Taka	1.03 (0.40–2.65)	0.948	0.63 (0.13–3.07)	0.570
100-10000Taka (1-101USD)	1.66 (0.67–4.07)	0.269	0.90 (0.16–5.12)	0.910
Place of residence				
Urban (ref)	1			
Semi-urban	1.05 (0.29–3.88)	0.93	0.63 (0.12–3.30)	0.584
Rural	1.79 (0.84–3.81)	0.13	1.60 (0.46–5.52)	0.455
Clinical diagnosis				
Autism Spectrum Disorders (ref)	1			
Down Syndrome	0.54 (0.14–2.15)	0.385	0.61 (0.12–3.09)	0.555
Cerebral Palsy	1.68 (0.81–3.48)	0.164	2.39 (0.83–6.87)	0.106

*Sig (*p* < 0.05)

## Discussion

Malnutrition is considered an important risk factor that can have a negative impact on the prognosis of patients with chronic neurological disease [19]. If malnutrition is not treated in time, it may impair the immune system [20] and aggravate neuromuscular disabilities [21]. Therefore, early identification of malnutrition in children with NDDs is important to ensure timely intervention, and to improve outcomes. Research confirms that children with disabilities are more likely to suffer from malnutrition, much more likely to be underweight or stunted [8, 10, 17]. The causal relationship between NDDs and malnutrition is bidirectional. Crepin et al.‘s research is an example of studies examining the biochemical link between malnutrition and epilepsy, for example, finding that protein-energy malnutrition or a lack of a few specific micronutrients in early childhood can affect the normal functioning of the Central Nervous System (CNS) and may be linked to the development of epilepsy in children [22].

This present study takes a different approach, aiming to explore malnutrition in a developing world hospital setting, where socioeconomic and cultural factors may significantly determine availability and provision of nutrition, working with a sample where disability in itself did not prevent adequate maintenance of food intake. By focusing on children who did not exhibit symptoms that should impede nourishment, the study allows a focus on the socioeconomic factors that may contribute to malnourishment. The result of this study showed that 58.6% of children in this category exhibited malnutrition, with a ratio of undernutrition to overnutrition of 4.4 to 1. There were significant negative associations between malnutrition and parental education level, in particular mother’s education level, fathers’ employment, urban residency, and monthly family income. The findings regarding education align with those of a very recent study conducted in Bangladesh, which discovered that children of mothers who completed their secondary or higher education were 1.77 and 1.50 times more likely, respectively, to meet developmental milestones than children of mothers who only completed their elementary education [23]. 

Collectively, the results suggest that malnourishment is likely mediated and moderated by socioeconomic factors, supporting other recent studies in this regard [24]. It is more pronounced for example in families with lower levels of education (particularly on the mother’s side) suggesting a role for health awareness education in improving outcomes. It is more pronounced in families living in rural areas, where access to health care is limited. Education and family location are thus likely mediating factors. The clearest likely moderating factor is household income. As the modal child in this study belonged to rural-based family with a monthly income below 10,000 BDT, access to adequate nourishment is a household problem. A high proportion of the household budget needs to be allocated to food, and food inflation in recent years has had a disproportionate effect on malnutrition in developing countries [25]. Nevertheless, poverty and lack of access to healthcare and public health advice and even higher quality education resources may have been confounded in the study, as all are associated with rural locations in Bangladesh. A longitudinal study of greater power may be able to disentangle the causal paths.

Interestingly, the current study of 157 children with NDDs showed an uneven gender split: there was a higher prevalence of NDDs in boys compared to girls, with a ratio of 1.13:1.00, with gender differences in cerebral palsy [26] being frequently observed. Few studies, particularly in LMICs, explore the role of gender in malnutrition among children with disabilities [8]. 

In our study, malnutrition was found to common in a range of conditions in which it is not inevitable. Even in the case of cerebral palsy (where in excess of 60% of the current sample exhibited malnutrition), clinicians now accept that under- or over-nutrition can be effectively addressed. Children with nervous system or brain problems such as those with cerebral palsy, Prader-Willi syndrome, Down syndrome, spina bifida, Rett syndrome, cognitive disabilities, developmental delays, ASD, and ADHD, experience obesity and overweight more severely [27]. 

The prevalence of obesity varies between 9.7% [28–30] and 35.7% [27] although in the current sample the proportion was much lower. Since it restricts their capacity to move independently, impairs their ability to breathe, and increases their risk of developing cardiovascular issues, obesity has always been a serious concern for children with NDD. For example, obesity in children with Down syndrome is harmful because it predicts adult obesity and the subsequent noncommunicable consequences like diabetes and hypertension [20, 27]. Individuals with ASD may suffer more from feeding problems and obesity compared to their peers as they have a specific preference towards sweet, cereal-based products and high protein and high fat foods. They also have a routine in their diet, which likely expands the tendency to have an unbalanced diet [31]. 

### Strength and limitations

While relatively novel in the developing world context in that it used hospital clinicians to conduct key components of this study, the sample size was relatively small, and cannot be extrapolated onto a national scale, particularly as the setting of the hospital chosen for this study is considered one of the relatively more prosperous districts in Bangladesh [32].

The study is also correlational, hence it cannot convincingly demonstrate causal direction. It is possible that family socioeconomic status is impacted by the presence of a child with disability., The burden of looking after a disabled child has been associated with impact on the family budget in previous studies [33]. and this may be particularly pronounced in a developing world setting.

There were also limitations in the measurement approach; simpler measures were chosen for analysing malnutrition and stunting, measures that are common to such studies but still not gold standard approaches. We incorporated BMI for age (BAZ) for 0–19 years to assess wasting or undernutrition in our research sample because WHO AnthroPlus only calculates WAZ for children aged 0-121 months; we were unable to determine the existence of general malnutrition (WAZ SD). Although peripheral blood film (PBF) and a complete blood count (CBC) may be used to identify iron deficiency, these invasive techniques are not usually used for this condition [34]. 

This study does, however, offer plausible evidence that socioeconomic status does impact on malnourishment outcomes in disabled children in Bangladesh. By excluding children suffering from conditions known to impact nutritional status, such as congenital heart disease, chronic renal disease and cleft lip, we were able to more clearly isolate how socioeconomic factors impact on malnourishment. It is nevertheless possible that dietary factors and oromotor dysfunction are significantly responsible for some of the malnutrition we observed in the sample.

**Conclusion**: The study results provide evidence of the impact of socio-demographic factors on the malnourishment of disabled Bangladeshi children. Parental education level, father’s occupation, and monthly family income all appear to be significantly related to the nutritional status of children with NDD, and cases of malnourishment appear more pronounced in children from rural areas. In absolute terms, the level of malnutrition found in the current sample is concerning. Amongst those children with cerebral palsy, in excess of 60% presented with undernutrition, a far higher percentage than is common in developed world hospital samples [35]. In the United States, where obesity is the most common form of malnutrition, even in a cerebral palsy hospital sample, obesity levels were 16.5% whereas in this study 11.8% which was positively related to the degree of disability in CP patients [36]. Child malnutrition in Bangladesh remains an issue. A recent study using The Multiple Indicator Cluster Survey (MICS) 2019 found that 24.67% of children are stunted, 9.75% are wasted, and 6.8% are overweight [37]. By comparison this hospital study, drawing from a population in a relatively prosperous district of Bangladesh, showed that 47.8% with stunting, and just 10.8% with overnutrition. The lower obesity rate is mildly encouraging; the much higher under nutrition scores are highly concerning. This study confirms that undernutrition is related to poverty, not an inevitable consequence of severe disability. More research is clearly warranted on the long-term consequences of malnutrition for children with a disability in the developing world.

### Recommendations

1. Children with NDD should be regularly screened for malnutrition in Bangladesh. This is particularly recommended in cases where physical factors impede feeding, or promote choking, regurgitation, and spilling of food.
2. Mass screening programmes should particularly focus on remote regions, and regions known to be economically disadvantaged. Expanding access to affordable health care for families with disabled children and offering nutritional advice and support will help reduce the prevalence of malnourishment.
3. Public health education programs focused on parents, offering advice on care and nutrition for their children may exert a positive influence on disabled children’s health and survival in Bangladesh.

### Electronic supplementary material

Below is the link to the electronic supplementary material.

Supplementary Material 1

## Data Availability

All the raw de-identified data will be made available upon reasonable request from the corresponding author.

## Footnotes

1 By comparison, media reports note that the Bangladesh labour ministry in 2023 proposed a minimum wage of 12,500 Bangladesh taka per month. This suggests that a significantly higher proportion of the families in this sample were living below the poverty line than the World Bank estimate at the time (24.3%).

## Abbreviations

ASD: Autism Spectrum Disorder
ADHD: Attention-Deficit / Hyperactivity Disorder
BAZ: BMI for Age Z Score
BMI: Body Mass Index
CBC: Complete Blood Count
CNS: Central Nervous System
CP: Cerebral Palsy
HIV: Human Immunodeficiency Virus
IPD: Indoor Patient Department
MAM: Moderate Acute Malnutrition
NDD: Neuro Developmental Disorder
OPD: Outdoor Patient Department
PBF: Peripheral Blood Film
SAM: Severe Acute Malnutrition
TMSS: Thengamara Mohila Sabuj Sangha
TMC: TMSS Medical College
TGHS: TMSS Grand Health Sector
TMC&RCH: TMSS Medical College & Rafatulla Community Hospital
WAZ: Weight for Age Z-Score
WHZ: Weight-for-Height Z-Score
WHO: World Health Organization
