# Supplementary material for: Nutritional status of children with neurodevelopmental disorders: a cross-sectional study at a tertiary-level hospital in northern Bangladesh
Source: BMC Nutr. 2024 Apr 19;10:61. doi: 10.1186/s40795-024-00863-9 (PMC11027387; doi:10.1186/s40795-024-00863-9)
Supplement: Supplementary file 1 — Supplementary Material 1 [file 40795_2024_863_MOESM1_ESM.doc]

**Consent Form**

1. **Protocol ID:**
2. **Patient ID:**
3. **Title of the study :** Nutritional status of children with neurodevelopmental disorders: A cross-sectional study at the tertiary level hospital in northern Bangladesh
4. **Principal Investigator :** Dr. Rabeya Khatun
5. **Institution:** TMSS medical College and Rafatullah Community Hospital,Thengamara,Bogura-5800.
6. **Purpose of the study:** To assess the sociodemographic profile and nutritional status of children with neurodevelopmental disorders.
7. **Data collecting process :**

Step-1: Interview

Step-2: Weight and height measuring

1. **Selection of the participant:** way of collecting data

Your child is chosen for this particular research or study no commendable research has yet been carried out in our Hospital.

1. **Expectation from and involvement of the participant:** You are kindly expected to give details history of your child’s illnesses and allow us to perform thorough clinical examination. We are looking forward to your co-operation & I request you, to stay in touch with us during the study period.
2. **Risk and benefit:**

There is no risk of your children in this study.

1. **Privacy, anonymity and confidentiality:**

You are ensured that all information should be kept confidential and you are liable to chose time and place which will be suitable for you.

1. **Right to withdraw:**

You are free to take part or refuse any part of the study.

If you are agreeing to our proposal of enrolling you / your patient in our study, please indicate that by putting your signature or your left thumb impression at the specified below.

Thank you for your co-operation

------------------------------------------------ ----------------------------------------------

Signature or left thumb impression of patient Signature or left thumb impression of patient

----------------------------------------------------- ----------------------------------------------

Signature or left thumb impression of witness Signature of investigator

Date------------------------

**Survey Questionnaire**

**Nutritional status of children with neurodevelopmental disorders: A cross-sectional study at the tertiary level hospital in northern Bangladesh**

**Questionnaire Seria**l Number: Date of Interview:

| **Section A: Bio-demographic Data of Child** | |
| --- | --- |
| **1. Name of the child:** | |
| **2. Age:** Date of Birth………./Month………/Year……… | |
| **3. Sex:** 1. Male 2. Female 3. Others | |
| **4. Educational Enrollment (5-12 Years)** | |
|  | 1. Yes 2. No |
| **5. Clinical Diagnosis** | |
|  | 1.Cerebral Palsy (CP) 2.CP with Epilepsy 3.Epilepsy 4.Down Syndrome  5. Down syndrome with Epilepsy 6.Autism Spectrum Disorder (ASD)  7.ADHD 8.Speech Delay 9.Developmental Delay 10.Intellectual Disability |

| **Section-B: Information of Parents** | |
| --- | --- |
| **1. Parent/guardian’s mobile no:** | |
| **2. Level of Education of Parents:** | |
| **2.1 Father’s Education Level** | |
|  | 1. No Formal Education 2. Up to Primary 3. Below SSC 4. SSC 5. HSC  6. Honors (Graduation) 7. Masters and Above |
| **2.1 Mother’s Education Level** | |
|  | 1. No Formal Education 2. Up to Primary 3. Below SSC 4. SSC 5. HSC  6. Honors (Graduation) 7. Masters and Above |
| **3. Occupation of the Parents** | |
| **3.1 Father’s Occupation** | |
|  | 1. Govt. service 2. Other service 3. Business 4. Day labor 5. Farmer 6. Unemployed 7.Others |
| **3.2 Mother’s Occupation** | |
|  | 1. Govt. service 2. Other service 3. Business 4. Day labor  5. Farmer 6. Unemployed 7.House wife 8. Others |
| **4. Area of Residence** | |
|  | 1. Urban 2. Semi urban 3. Rural |
| **5. Family Income:** | |
|  | 1. <10000 Taka 2. 11000-25000 taka 3. 26000-35000 taka 4.>35000 taka |
| **6. Family Types** | |
|  | 1.Nuclear Family 2.Joint/Extended Family |
| **7. Household Size** | |
|  | 1. 1-3 Members (Small Family) 2. 4-5 Members (Medium Family)  3. >6 Members (Large Family) |
| **Section-C: Mother’s gestational history** | |
| **1. H/O allergic reaction to any food-** | |
|  | 1. Yes 2. No |
| **2. Avoid any food during pregnancy** | |
|  | 1. Yes 2. No |
| **3. History of any illness/disease during pregnancy-** | |
|  | 1.GDM 2.HTN 3.Chronic Renal disease 4.Others 5.No history of such illness |
| **4. Mode of delivery** | |
|  | 1.C/S 2. NVD |
| **Section-D: Post natal history of child** | |
| **1.After delivery Colostrums given** | |
|  | 1. Yes 2. No |
| **2. Starts Breast feeding within 1 hour of delivery-** | |
|  | 1. Yes 2. No |
| **3. Practiced EBF-** | |
|  | 1. Yes 2. No |
| **4. Does the child have any history of following illness after birth-** | |
|  | 1. CHD 2. Kidney disease 3. Cleft lip & Palate 4. Birth Asphyxia  5. Kernicterus 6. Premature baby 7. No history of such illness 8.Other |

| **Section-E: clinical (Neurological and nutritional) Finding of Children** | |
| --- | --- |
| **1.Neurological /physical finding of children** | |
| **1.1 Does the child have abnormal/involuntary muscle movement/slow repetitive movement of hands, neck (Dyskinesia)?** | |
|  | 1. Yes 2. No |
| **1.2.Does the child have abnormal posture/muscle spasm/twisting of neck (Dystonia)?** | |
|  | 1. Yes 2. No |
| **1.3.Does the child have restlessness / hyperactivity?** | |
|  | 1. Yes 2. No |
| **1.4.Does the child have Seizure?** | |
|  | 1. Yes 2. No |
| **1.5. GMFC System classification according to age of the child** | |
|  | 1.Level I 2. L-II 3. L- III 4. L-IV 5. L-V 6.None |
| **2.Nutritional status finding of children** | |
| **2.1.Visible severe wasting** | |
|  | 1. Yes 2. No |
| **2.2. Bipedal pitting edema (6 months to 59 months)-** | |
|  | 1. Yes 2. No |

| **Section-F: factors causing feeding Difficulties** | |
| --- | --- |
| **1.Oro-motor Dysfunction** | |
| **1.1 Does the child have history of poor suckling effort?** | |
|  | 1. Yes 2. No |
| **1.2 Does the child choke/cough while feeding?** | |
|  | 1. Yes 2. No |
| **2. Loss of Foods** | |
| **2.1 History of regurgitation of food or vomiting after feeding** | |
|  | 1. Yes 2. No |
| **2.2 History of spilling the food while feeding** | |
|  | 1. Yes 2. No |
| **3. Inadequate food intake due to-** | |
| **3.1 Oral sore-** | |
|  | 1. Yes 2. No |
| **3.2 Dental caries-** | |
|  | 1. Yes 2. No |

| **Section-G: Dietary History of child** | |
| --- | --- |
| **1.Types of Food Provided to Children** | |
| **1.1 Texture of food** | |
|  | 1.Solid form 2.Semi-solid form 3.Blended /liquid form 4.Semi-solid & blended |
| **1.2Types of Diet given** | |
|  | 1.Breast milk 2. Formula milk 3. Breast milk+ Formula milk 4.Breast milk+Khichury 5.Formula milk+Khichury 6.Milk+ Suji/rice flour/Cerelac 7.Suji 8.Khichury 9.Cow milk 10.All types of foods/Family foods 11.Others |
| **2. Foods intake in last 24 hours** | |
| **2.1 Numbers of Meals per day/ Frequency of meal in 24 hours** | |
|  | 1. 3 Meals & 2 Snacks 2. 3 Meals 3. 2 Meals 4. Once |
| - 1. **Numbers of meals constituting Balance Diet in 24 hours** | |
|  | 1. None 2. One 3. Two 4. Three |
| **3. Duration taken while Feeding** | |
|  | 1.20 minutes 2. > 20 minutes |
|  | |
| **Section-H: Effect of Rehabilitation(Nutritional & Physical) on nutritional status of child** | |
| **1. Does the child get any specific feeding aid? eg- Special spoon, glass, sitting chair etc.** | |
|  | 1. Yes 2. No |
| **2. Does the mother receive any nutritional counseling as a part of child management?** | |
|  | 1. Yes 2. No |
| **3. How long the children have been on occupational therapy/developmental therapy?** | |
|  | 1. ≤ 3 months 2. 3-6 months 3. >6 months 4. None |

| **Section-II : Anthropometric Measurement of the Child** | | | | | | |  |
| --- | --- | --- | --- | --- | --- | --- | --- |
| **A. Child < 5 years** | | | **B. Child ˃5 years** | | | |  |
| Weight in Kg | : |  | | Weight in Kg | : |  | |
| Height /Length/Tibial Length in cm | : |  | | Height /Length/Tibial Length in cm | : |  | |
| OFC | : |  | | BMI | : |  | |
| MUAC(>6month) | : |  | |  | : |  | |

**THANK YOU FOR YOUR VALUABLE ANSWERS**
